# Bridging the gap between guidelines and practice: genetic counseling uptake and barriers in patients with ovarian malignancies

**DOI:** 10.1186/s12905-026-04940-y

**Published:** 2026-09-29

**Authors:** Walid Shaalan, Nicola Dikow, Felix K. F. Kommoss, Katharina Smetanay, Nourhan Hassan, Maximilian Riedel, Kyra Fischer, Friederike Stroisch, Cecilie F. Torkildsen, Foteini Katsikogianni, Lara Meike Tretschock, Kathrin Hassdenteufel, Dina Batarseh, Riku Togawa, Christina Schmidt, Osama Azzam Nieto, Thomas M. Deutsch, Axel Gerhardt, Andre Hennigs, Lisa Katharina Nees, Steffen Hirsch, Andreas Schneeweiss, Oliver Zivanovic, Fabian Riedel

**Affiliations:** 1 Department of Gynecology and Gynecological Oncology, Heidelberg University Hospital, Im Neuenheimer Feld 440, Heidelberg, 69120 Germany; 2 Institute of Human Genetics, University Hospital Heidelberg, Im Neuenheimer Feld 440, Heidelberg, 69120 Germany; 3 Institute of Pathology, University Hospital Heidelberg, Im Neuenheimer Feld 224, Heidelberg, 69120 Germany; 4 National Center for Tumor Disease (NCT), University Hospital Heidelberg, Im Neuenheimer Feld 460, Heidelberg, 69120 Germany; 5 Center for Molecular Medicine Cologne, University of Cologne, Robert- Koch-Street 21, Cologne, 50931 Germany; 6 Department of Obstetrics and Gynecology, School of Medicine and Health, Klinikum rechts der Isar, Technical University of Munich, Ismaninger Street 22, Munich, 81675 Germany; 7 Department of Clinical Science, Centre for Cancer Biomarkers CCBIO, University of Bergen, Bergen, 5020 Norway

**Keywords:** Ovarian malignancy, Genetic counseling, Germline genetic testing, Patient reported outcomes, Problem frequency framework

## Abstract

**Background:**

Genetic counseling and germline genetic testing are essential components of ovarian cancer care; however, their implementation in routine clinical practice remains inconsistent. This study aimed to evaluate the utilization of genetic counseling and testing among patients with ovarian cancer and identify barriers to implementation within routine care.

**Methods:**

A single-center, retrospective questionnaire-based study was conducted among patients who underwent surgical treatment for ovarian malignancies between January 2021 and June 2025. Patient-reported data on information provision, referral pathways, organizational access, satisfaction, and barriers were linked with clinical and demographic characteristics. Descriptive analyses were performed, reflecting the exploratory nature of the study. A problem frequency (PF) framework, defined as the proportion of responses indicating a problem, was applied to identify and rank patient-reported barriers.

**Results:**

Of 321 identified patients, 61 were deceased prior to study initiation. Among 256 successfully contacted patients, 132 responded (51.6%). Overall, 115/132 (87.1%) reported receiving information regarding genetic counseling, primarily from hospital-based physicians (106/132; 80.3%). Among the 132 questionnaire respondents, 96/132 (72.7%) attended genetic counseling and 93/132 (70.5%) proceeded to genetic testing; pathogenic variants were identified in 26/93 tested patients (28.0%). Genetic counseling among respondents increased from 37.5% in 2021 to 94.7% in 2025. The majority of appointments were self-initiated. The most frequently reported barriers were limited information provision, appointment scheduling difficulties, and travel-related barriers, whereas emotional burden at the time of diagnosis was reported less frequently. Uptake was significantly associated with family history of breast and/or ovarian cancer, higher educational attainment, and parity. Notably, nearly half of the participants who had not attended genetic counseling (17/36;47.2%) reported reconsidering uptake after completing the questionnaire.

**Conclusion:**

Despite recommendations for universal genetic counseling in ovarian cancer care, gaps in implementation remain evident in routine clinical practice. Organizational and informational barriers represent prominent obstacles to uptake. Our findings highlight the potential value of standardized referral pathways, proactive information provision, and repeated patient engagement to facilitate access to genetic services.

**Graphical Abstract:**

**Figure:**
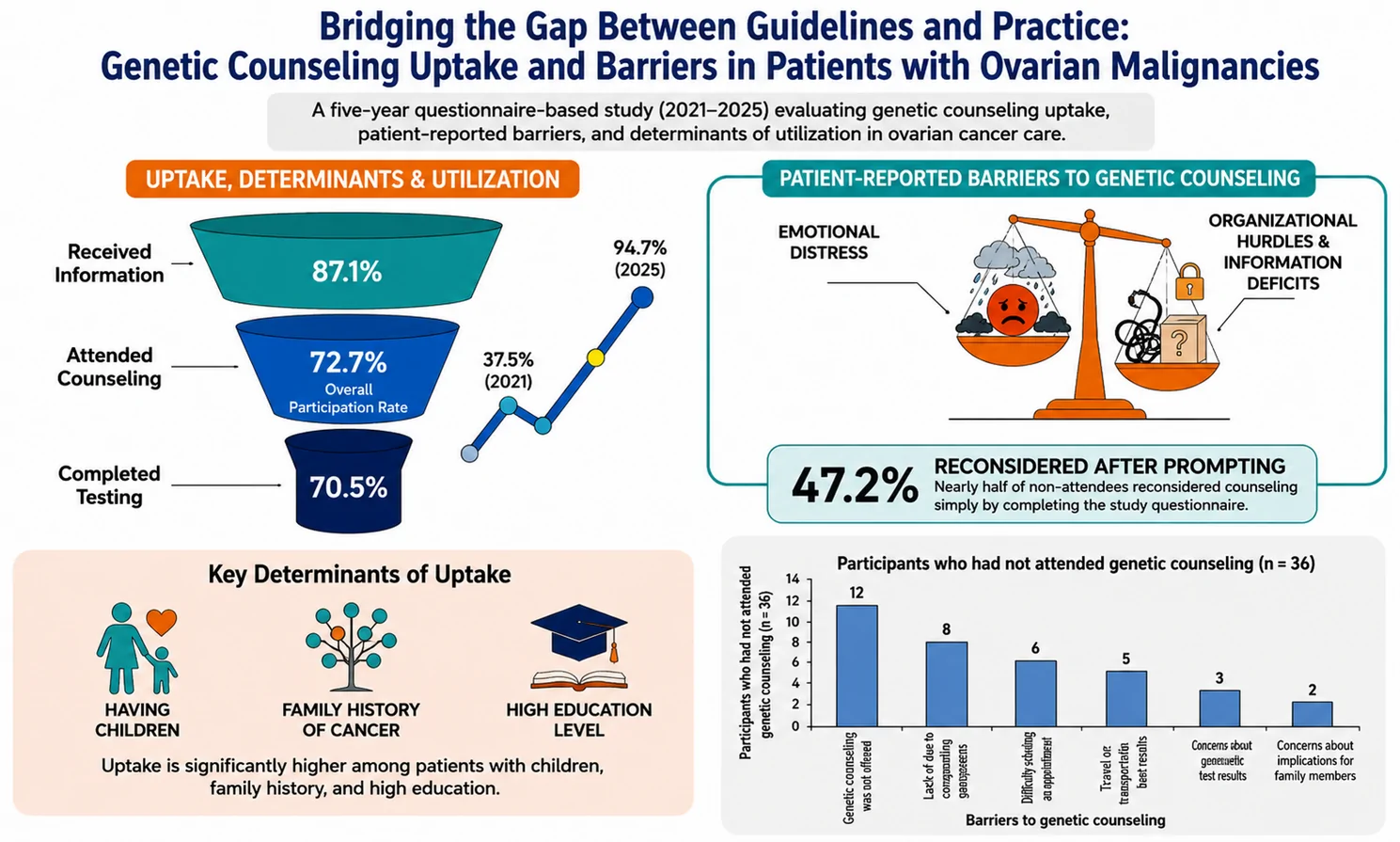

**Supplementary Information:**

The online version contains supplementary material available at https://doi.org/10.1186/s12905-026-04940-y.

## Background

Ovarian cancer ranks as the leading cause of death among gynecologic malignancies worldwide and continues to pose a major clinical and public health challenge due to its frequently late diagnosis, aggressive biological behavior, and limited effectiveness of screening strategies [1, 2]. In Germany, approximately 6,700 women are newly diagnosed with ovarian cancer each year, and more than two thirds with advanced FIGO (International Federation of Gynecology and Obstetrics) stage III or IV disease at initial diagnosis. Advanced stages are associated with five-year overall survival rates of 40–45%, compared with survival rates exceeding 90% in patients diagnosed at an early stage [1, 3]. These unfavourable survival outcomes underscore the need for risk-adapted prevention strategies and individualized therapeutic approaches [2, 4].

A substantial proportion of ovarian malignancies arise in the context of hereditary cancer predisposition syndromes. Germline pathogenic variants in *BRCA1* and *BRCA2* (Breast cancer gene) account for approximately 10–20% of epithelial ovarian cancers, while additional susceptibility genes, including *PALB2*,* RAD51C*,* RAD51D*,* BRIP1* and mismatch repair genes associated with Lynch syndrome, further contribute to hereditary Risk [5, 6]. Importantly, several studies have demonstrated that a considerable proportion of variant carriers would be missed if genetic testing were restricted to patients selected on the basis of young age at diagnosis or a positive family history alone, thereby supporting the paradigm shift toward universal genetic counseling and testing for patients with epithelial ovarian cancer. Genetic services for hereditary susceptibility to cancer can prevent many cancer diagnoses and deaths through identification of genetic cancer predisposition syndromes in affected individuals, early identification of their relatives at risk and risk management strategies such as surveillance or risk reducing surgery [6–8].

Beyond its relevance for risk stratification and cancer prevention in relatives, the identification of germline and somatic alterations in homologous recombination repair pathways has become highly relevant for treatment allocation. *BRCA*-mutated and homologous recombination-deficient (HRD) tumors exhibit increased sensitivity to platinum-based chemotherapy and Poly ADP-Ribose Polymerase (PARP) inhibitors, which has translated into clinically significant improvements in progression-free and overall survival in both recurrent and maintenance treatment strategies [2, 9]. Consequently, genetic information has evolved from a preventive tool to a central component of contemporary precision oncology in ovarian cancer [9].

Accordingly, the German guidelines on malignant ovarian tumors recommend that all patients with ovarian cancer should be offered structured genetic counseling and, where appropriate, germline genetic testing, irrespective of age at diagnosis, histologic subtype, or family history [10]. In Germany, the provision of molecular germline analyses is embedded within a clearly defined framework under the Genetic Diagnosis Act (Gendiagnostikgesetz, GenDG), which traditionally requires specialized genetic counseling [11]. More recently, a shift toward mainstreaming genetic testing has emerged, enabling oncologists to initiate testing directly in order to facilitate timely access to genetic services, while the German Consortium for Hereditary Breast and Ovarian Cancer (GC-HBOC) continues to define national standards of care within specialized hereditary cancer networks [12].

Despite these well-established recommendations and regulatory structures, the uptake of genetic counseling in the routine clinical practice remains inconsistent. Real-world data from specialized oncologic centers indicate that referral and utilization rates of genetic counseling fall substantially below guideline recommendations, reflecting persistent gaps in implementation [6, 7] Qualitative and quantitative studies have identified multifactorial barriers, including limited awareness of genetic counseling, insufficient communication by health care professionals, organizational constraints, emotional burden at the time of diagnosis and throughout the treatment process, as well as concerns regarding familial implications of genetic test results [4, 6].

Furthermore, patient preferences regarding the timing of genetic counseling vary widely across the disease trajectory, emphasizing that standardized referral pathways need to be complemented by flexible, patient-centered approaches [9].

Against this background, there is a clear need for context-specific data on the real-world utilization of genetic services in different healthcare systems. The acceptance rate of genetic counseling is variable, ranging from 60 to 99% [13–16]. In Germany, systematic evidence on the uptake of genetic counseling among ovarian cancer patients remains limited [11].

The present questionnaire-based study aimed to assess the uptake of genetic counseling among patients with ovarian cancer treated at a university women´s hospital. By integrating patient-reported experiences with clinical and demographic characteristics, the study further sought to identify determinants of counseling uptake, characterize barriers to accessing genetic counseling, and evaluate challenges in the implementation of guideline-concordant genetic services in routine ovarian cancer care.

## Methods

### Study design

In this monocentric study, we identified all patients who underwent surgical treatment for ovarian malignancies at the Department of Gynecological Oncology, Heidelberg University Hospital, between January 1. ,2021, and June 30. ,2025. Patients who were deceased at the time of study initiation, patients whose study invitations were returned as undeliverable and patients younger than 18 years were excluded from the analysis. The study was conducted in accordance with the Declaration of Helsinki and received approval from the Institutional Review Board at Heidelberg University Hospital (IRB Approval Number: S-155/2025, date: 22.04.2025). A written informed consent was obtained from all participating patients.

### Recruitment and data collection

Data were obtained from dual data sources, comprising institutional hospital information system records and patient-completed questionnaires. Eligible patients were identified through the institutional hospital information system and surgical records. Potential participants were contacted by postal mail and invited to take part in the study. The study package comprised an invitation letter, written study information, an informed consent form, and a standardized questionnaire in the German language. Participants were requested to return the signed informed consent form together with the completed questionnaire by mail using the provided prepaid return envelope. In case of non-response, a single reminder was sent to patients who had not declined participation 1 month after the initial invitation.

Clinical and demographic data were extracted from the electronic medical records of consenting patients, including age at the time of diagnosis, Eastern Cooperative Oncology Group (ECOG) performance status at diagnosis, FIGO stage, histologic subtype, use of neoadjuvant and/or adjuvant chemotherapy, disease recurrence and date of surgery.

### Instrumentation

While the questionnaire was specifically developed by a multidisciplinary team of oncologists and geneticists for this study and had not undergone previous validation [2], its items were derived from established patient-reported outcome principles [17, 18]. To support face validity and clarity, a pilot phase was conducted with five patients prior to study initiation. Genetic counseling attendance was defined as participation in at least one consultation with a qualified genetics professional prior to or in conjunction with genetic testing [19]. Genetic counseling was provided by physician specialized in human genetics at the Institute of Human Genetics, Heidelberg University Hospital, or by corresponding specialists at external genetic centers. Treating physicians or healthcare providers informed patients about the recommendation for genetic counseling and facilitated referral to genetic services. Germline genetic testing was performed using the TruRisk^®^ gene panel of the German Consortium for Hereditary Breast and Ovarian Cancer, comprising *BRCA1/2* and additional hereditary cancer susceptibility genes [20].

The questionnaire consisted of four sections. Section  1 assessed whether participants had been informed about the recommendation for genetic counseling and genetic testing as part of their oncologic treatment. Information coverage included whether and when patients learned about the role of genetic counseling and by whom this information was provided. Section  2 addressed referral pathways and organizational aspects, including those who initiated the referral, whether counseling and/or testing were undertaken, where counseling was provided, and organizational factors including travel mode, and sources of preparatory information. For participants who did not undergo genetic counseling or testing, reasons for non-attendance as well as post-survey reconsideration were assessed. Section  3 evaluated patient satisfaction with the information received and waiting times using 1–10 numeric rating scales, as well as perceived barriers and facilitators to utilization, and the perceived relevance of genetic testing for personal medical care and implications for family members. Section  4 collected sociodemographic and clinical characteristics, including family history of breast and/or ovarian cancer, parity, native language, employment status and level of education.

### Statistical analysis

Statistical analyses were primarily descriptive, reflecting the exploratory nature of the study. Categorical variables were summarized using absolute frequencies and percentages, whereas continuous variables were reported as medians and interquartile ranges (IQRs). Missing data were analyzed descriptively, and no imputation procedures were performed. Inferential statistics were additionally applied to identify potential sociodemographic associations that may warrant further prospective investigation. Given the exploratory study design, no adjustment for multiple testing was performed. Data were analyzed using IBM SPSS software version 20.0 (Armonk, NY, USA: IBM Corp.) and GraphPad Prism version 10.0. Statistical significance was set at *p*-value < 0.05.

## Results

Between January 1, 2021, and June 30, 2025, a total of 321 patients who underwent surgical treatment for ovarian malignancies at the Department of Gynecological Oncology, Heidelberg University Hospital were identified. Of these, 61/321 patients (19%) in the total cohort were documented to have died prior to study initiation and were excluded. The remaining 260 eligible patients met the inclusion criteria and were invited to participate. Study invitations were returned as undeliverable in 4/260 patients (1.5%) due to invalid or outdated contact information; these patients were excluded from further analyses. A total of 132 of 256 patients returned the questionnaire, corresponding to a response rate of 51.6%. Responders and non-responders did not differ significantly with regard to age, ECOG performance status, histological subtype or FIGO stage.

Among respondents (*n* = 132), the median age was 60.0 years (IQR 50.5–68.5), with the majority (101/132; 76.5%) exhibiting an ECOG performance status of 0. High-grade serous ovarian carcinoma (HGSOC) was the predominant histologic subtype (83/132;62.9%) and 82/132 (62.1%) patients presented with advanced-stage disease (FIGO III-IV). Regarding therapeutic management, 40/132 (30.3%) patients underwent neoadjuvant chemotherapy, while 65/132 (49.2%) received adjuvant chemotherapy. Disease recurrence was documented in 10/132 (7.6%) patients (Table 1).

**Table 1:** Patient demographic and clinical characteristics (*n* = 132)

Age (years)		
Minimum – Maximum	19.0–89.0	
Median	60.0	
IQR	50.50–68.50	
ECOG status at diagnosis	(*n*)	(%)
0	101	(76.5%)
1	27	(20.5%)
2	4	(3.0%)
FIGO Stage		
I and II	40	(30.3%)
III and IV	82	(62.1%)
Recurrence	10	(7.6%)
Histopathological subtype		
High-grade serous	83	(62.9%)
Low-grade serous	8	(6.1%)
Borderline	13	(9.8%)
Endometrioid	9	(6.8%)
Granulosa cell	6	(4.5%)
Clear cell	6	(4.5%)
Mucinous	4	(3.0%)
Other rare tumors	3	(2.3%)
Chemotherapy status		
Neoadjuvant chemotherapy	40	(30.3%)
Adjuvant	65	(49.2%)
No chemotherapy	27	(20.5%)

Overall, 115/132 respondents (87.1%) reported having received information about the recommendation for genetic counseling and genetic testing as part of their oncologic treatment. Among these participants, the treating hospital physician (oncologic surgeon or medical oncologist) was the most frequently reported source of information (106/132; 80.3%). A smaller subset of respondents (*n* = 9) reported receiving information from other sources, including outpatient gynecologists (4/132; 3.0%), family physicians (3/132; 2.3%), or other healthcare providers (2/132; 1.5%). Conversely, 12/132 (9.1%) respondents reported not having been informed, and 5/132 (3.8%) could not recall. This distinction was based on patient questionnaire responses and documentation of recommendations in discharge records.

The timing of information provision varied across the treatment trajectory. Most patients reported receiving information in the postoperative period within 5 weeks after primary debulking surgery (47/115; 40.9%) or interval debulking surgery (37/115; 32.2%) [21]. Information was less frequently provided during chemotherapy (18/115; 15.7%) or after completion of chemotherapy (6/115; 5.2%). Seven patients (7/115; 6.1%) reported receiving information at other time points, in the context of disease recurrence or during subsequent follow-up (Table 2).

**Table 2:** Information provision, utilization, and organizational aspects of genetic counseling and testing

	Number	(%)	
1. Source of information about genetic counseling and testing recommendation (*n* = 132)			
Received information about genetic counseling/testing	115	87.1%	
└─ Treating hospital physician (oncologic surgeon or medical oncologist)	106	80.3%	
└─ Outpatient gynecologist	4	3.0%	
└─ Family physician	3	2.3%	
└─ Other healthcare provider	2	1.5%	
Not informed	12	9.1%	
Do not recall receiving information	5	3.8%	
2. Timing of first information about genetic counseling and testing (*n* = 115)			
Postoperative period after primary debulking surgery	47	40.9%	
Postoperative period after Interval debulking surgery	37	32.2%	
During chemotherapy	18	15.7%	
After completion of chemotherapy	6	5.2%	
At recurrence or disease progression	4	3.5%	
During the follow-up	3	2.6%	
3. Utilization of genetic counseling and testing (*n* = 132)			
Attended ≥ 1 genetic counseling visit	96	72.7%	
└─ Received germline genetic testing after counseling	93	70.5%	
└─Pathogenic variant	26/93	28.0%	
└─Variant of uncertain significance (VUS)	4/93	4.3%	
└─Negative	61/93	65.6%	
└─Result unavailable	2/93	2.2%	
Did not attend genetic counseling	36	27.3%	
4. Uptake of genetic counseling by year of surgery			
2021	6/16	37.5%	
2022	17/20	85%	
2023	21/28	75%	
2024	34/49	69.5%	
2025 (until 6/25)	18/19	94.7%	*p* = 0.002^*^
5. Factors influencing attendance at genetic counseling (*n* = 96)			
Recommendation by gynecologic surgeon	52	54.2%	
Recommendation by medical oncologist	27	28.1%	
Influence of relatives or friends	9	9.4%	
Recommendation by outpatient gynecologist	6	6.3%	
Recommendation by family physician or other healthcare provider	2	2.1%	
6. Initiation of the genetic counseling appointment (*n* = 96)			
Patient self-initiated	54	56.3%	
Healthcare professional (Pelvic Care Nurse)	21	21.9%	
Medical oncologist	11	11.5%	
Relatives or friends	10	10.4%	
7. Transportation to genetic counseling (*n* = 96)			
Private car	75	78.1%	
Public transport	12	12.5%	
Taxi	7	7.3%	
Do not recall	2	2.1%	
8. Place of genetic counseling (*n* = 96)			
Institute of Human Genetics of the university hospital	71	74.0%	
External specialized genetic center	25	26.0%	
9. Reconsideration of genetic counseling after completion of the questionnaire (*n* = 36)			
Yes	17	(47.2%)	
No	15	(41.7%)	
Undecided	4	(11.1%)	

Within the respondent cohort, 96/132 (72.7%) attended at least one genetic counseling visit, and 93/132 (70.5%) proceeded to genetic testing. Among those tested, pathogenic variants were identified in 26/93 (28.0%), most frequently involving *BRCA1* (12/26; 46.2%), *BRCA2* (5/26; 19.2%), and *BRIP1* (3/26; 11.5%); the remaining 6/26 (23.1%) involved other hereditary cancer susceptibility genes. Variants of uncertain significance (VUS) were identified in 4/93 (4.3%), results were unavailable in 2/93 (2.2%), and 61/93 (65.6%) tested negative. Genetic counseling uptake among respondents significantly increased over the study period (*p* = 0.002): 37.5% (6/16) in 2021, 85.0% (17/20) in 2022, 75.0% (21/28) in 2023, 69.4% (34/49) in 2024, and 94.7% (18/19) in 2025 (Table 2).

Among respondents who attended genetic counseling (*n* = 96), recommendations by the gynecologic surgeon were the most frequently cited factor influencing attendance (52/96; 54.2%), followed by recommendations from the medical oncologist (27/96; 28.1%). Influence from relatives or friends was reported by 9 participants (9/96; 9.4%). Smaller proportions attributed their decision to their outpatient gynecologist (6/96; 6.3%) or to their family physician or other healthcare providers (2/96; 2.1%). The arrangement of the first genetic counseling visit differed among participants. The majority of appointments were initiated by the patients themselves (54/96; 56.3%) after receiving the relevant contact information, followed by facilitation by healthcare professionals within the department, most commonly a pelvic care nurse (21/96; 19.8%). In a smaller proportion of patients, medical oncologists (11/96; 11.5%) and relatives or friends (10/96; 10.4%) were involved in initiating the appointment process. Participants reported using different means of transportation to attend the initial genetic counseling visit. The most commonly used mode of transport was a private car (75/96; 78.1%), followed by public transportation (12/96; 12.5%). A smaller proportion of respondents reported using a taxi (7/96; 7.3%), while a minority were unable to recall the mode of transportation (2/96; 2.1%). Genetic counseling was provided in different settings, predominantly at the Institute of Human Genetics of the University Hospital (71/96; 74.0%), with the remaining conducted at external specialized centers (25/96; 26.0%). Among the 36 participants who had not yet undergone counseling, 17/36 (47.2%) reported that the questionnaire promoted reconsideration of genetic counseling uptake. These data are listed in Table 2.

**Fig. 1:**
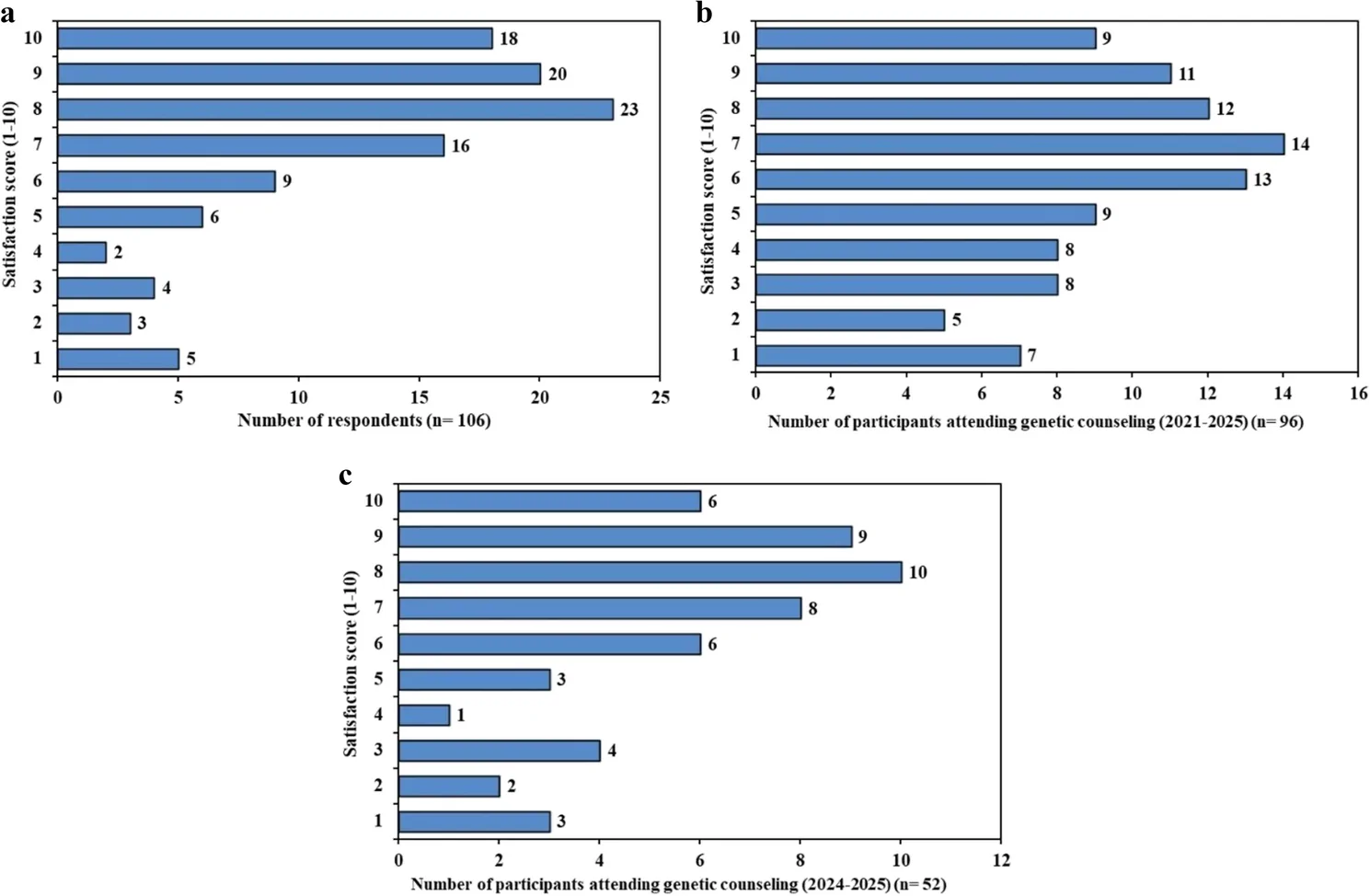
**a**: Distribution of patient-reported satisfaction scores regarding information provided about genetic counseling within the university hospital. Participants rated their satisfaction using a 1-10 numeric rating scale. Bars indicate the number of respondents for each score (*n*=106). **b**: Distribution of patient-reported satisfaction with waiting times for the initial genetic counseling appointment. Participants rated their satisfaction using a 1-10 numeric rating scale. Bars represent the number of participants reporting each score (*n*=96). Y- axis: Satisfaction score. X- axis: Number of participants attending genetic counseling (2021-2025). **c**: Distribution of patient-reported satisfaction with waiting times for the initial genetic counseling appointment in the year 2024 and 2025. Participants rated their satisfaction using a 1-10 numeric rating scale. Bars represent the number of participants reporting each score (*n*=52). Y- axis: Satisfaction score. X- axis: Number of participants attending genetic counseling (2024-2025)

Participants who had received information about genetic counseling within the university hospital rated their satisfaction with the information provided on a 1–10 numeric rating scale. Overall, 72.6% of respondents reported a satisfaction score of ≥ 7 (Fig. 1a).

Satisfaction with waiting times for the initial genetic counseling appointment varied widely across the total cohort, with higher satisfaction scores observed in the later study years 2024 and 2025, as shown in Fig. 1b and c.

**Fig. 2:**
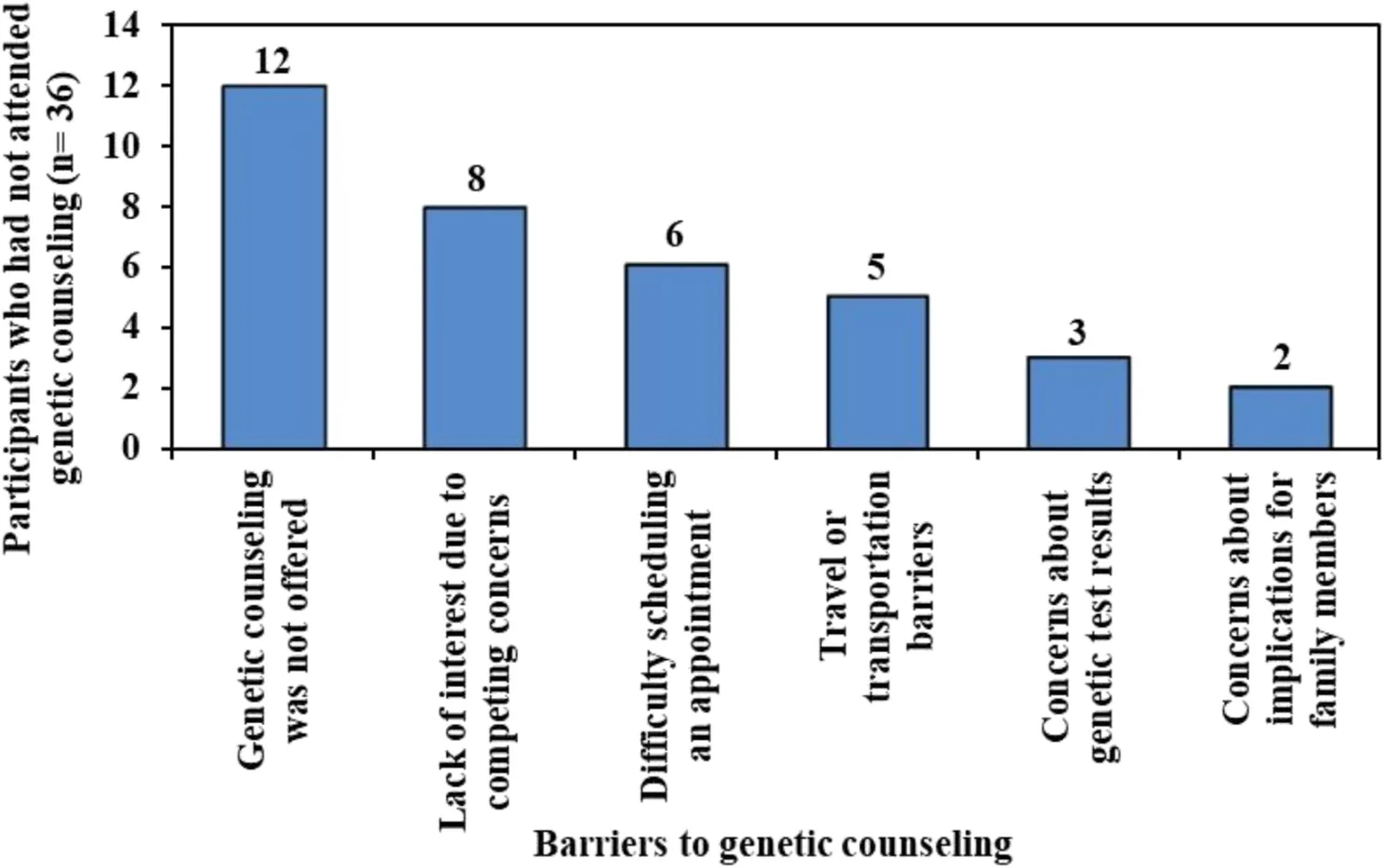
Distribution of patient-reported barriers to genetic counseling among participants who had not undergone genetic counseling. Bars represent the number of respondents reporting each barrier (*n*=36)

To provide a structured summary of patient-reported barriers among participants who had not undergone genetic counseling, the problem frequency was calculated [18]. Problem frequency reflected the proportion of responses indicating the presence of a problem. The problem frequency analysis demonstrated that the highest burden of reported problems clustered within domains related to deficits in information provision and organizational or logistical barriers to accessing genetic counseling. In contrast, domains related to emotional burden at the time of diagnosis exhibited comparatively lower problem frequency values (Fig. 2). Among participants who had not undergone genetic counseling (*n* = 36), the most frequently reported barriers were lack of referral or information provision (12/36; 33.3%), competing personal concerns (8/36; 22.2%), appointment scheduling difficulties (6/36; 16.7%), and travel or transportation barriers (5/36; 13.9%), whereas emotional concerns about genetic test results or familial implications were reported less frequently.

Uptake of genetic counseling was significantly associated with having children (77/96 vs. 21/36; *p* = 0.010), a positive family history of breast and/or ovarian cancer (50/96 vs. 10/36; *p* = 0.013), and higher educational attainment, defined as possession of a university entrance qualification (53/96 vs. 11/36; *p* = 0.012) [22]. No significant associations were found for employment status or native language (Table 3).

**Table 3:** Association between genetic counseling uptake and participants’ characteristics

Variable	Genetic Counseling (*n* = 96)	No genetic counseling (*n* = 36)	ꭓ^2^	*p* value
Parental status				
Has Children	77 (80.2%)	21 (58.3%)	6.551*	0.010^*^
No Children	19 (19.8%)	15 (41.7%)		
Family history of breast and/or ovarian cancer				
Yes	50 (52.1%)	10 (27.8%)	6.238^*^	0.013^*^
No	46 (47.9%)	26 (72.2%)		
Level of education				
High education ^a^	53 (55.2%)	11 (30.6%)	6.371^*^	0.012^*^
Medium or low education	43 (44.8%)	25 (69.4%)		
Native language				
German	78 (81.3%)	24 (66.7%)	3.171	0.075
Other	18 (18.8%)	12 (33.3%)		
Employment status				
Currently employed at Diagnosis	41 (42.7%)	17 (47.2%)	0.217	0.642
Not currently employed at Diagnosis ^b^	55 (57.3%)	19 (52.8%)		

^a^ Higher educational attainment was defined as possession of a university entrance qualification

^b^ Includes unemployed participants, retirees receiving pension and other non-working participants

χ^2^ Chi square test was used to assess associations between categorical variables and genetic counseling uptake

* Statistically significant at *p* ≤ 0.05

## Discussion

In this German single-center study, we evaluated the utilization and awareness of genetic counseling and testing among patients with ovarian cancer. Among survey respondents, our findings indicate a persistent gap between recommendations for universal genetic counseling and their implementations in routine clinical care. Both the national AGO guidelines (Arbeitsgemeinschaft Gynäkologische Onkologie) and the international guidelines from the European Society for Medical Oncology (ESMO) and the National Comprehensive Cancer Network (NCCN) recommend universal referral for genetic counseling and testing [10, 23, 24].

Although the majority of participants reported having received information regarding genetic counseling, substantial proportion either did not recall such information or reported not having been informed [25].

Similar challenges in implementing guideline recommended genetic assessment have been reported across different healthcare systems. In a systematic review and meta-analysis of 35 studies, Lin et al. reported pooled rates of 39% for referral to genetic counseling and 30% for completion of genetic testing in the absence of targeted interventions. Testing completion was higher in more contemporary cohorts, reaching approximately 63%, while targeted implementation strategies were associated with further improvements, with pooled completion rates of up to 99% for mainstream genetic testing [26]. Bokkers et al. similarly demonstrated that implementation of a mainstream testing pathway increased the proportion of newly diagnosed patients offered germline genetic testing from 56% to 70%, while reducing genetics-related healthcare costs by 31% [27]. More recently, Lanjouw et al. reported an overall *BRCA1/2* testing rate of 80.4%; importantly, most patients with unknown germline status had not been offered testing rather than having declined it [28]. These findings are consistent with our observation that informational and referral-related barriers were prominent among patients who had not attended genetic counseling. Alternative strategies, including tumor-first testing, may further improve the efficiency of hereditary cancer identification, although their impact on uptake depends on successful integration into clinical pathways [29]. Taken together, our counseling attendance of 72.7% and germline testing rate of 70.5% among survey respondents are within the range reported in contemporary clinical practice, while remaining below the goal of universal genetic assessment.

Beyond differences in overall uptake, previous studies have identified several patient- and care pathway-related barriers to genetic counseling. In a qualitative study by Vogel et al., insufficient awareness of genetic counseling, low perceived relevance, and logistical constraints were identified as important barriers to utilization [4]. Emotional burden at diagnosis has also been described as a relevant barrier to genetic counseling and testing, although this was less frequently reported as a dominant factor in our cohort [5].

To further characterize patient-reported barriers, a structured quantitative problem frequency approach was used to systematically aggregate and rank problem domains across the care pathway. Consistent with Kleeberg et al., domains related to information and organizational access emerged as the most prominent problem areas [18]. This may indicate that the relative importance of psychosocial versus organizational barriers varies across care settings and stages of treatment [5].

Conceptually, the problem frequency framework used in our study overlaps with domains of the Consolidated Framework for Implementation Research (CFIR), particularly those related to the inner setting and implementation processes [30]. In contrast to broader implementation frameworks such as RE-AIM, the problem frequency approach provides a more granular patient-centered perspective on specific barriers encountered during the clinical care pathway [31].

Furthermore, our findings suggest that the timing of information provision regarding genetic counseling may influence patient recall, highlighting the importance of repeated and reinforced communication throughout treatment and follow-up. In line with previous studies, we observed considerable variation in timing, ranging from early treatment phases to later follow-up or recurrence. Early integration of information regarding genetic counseling has been proposed to facilitate uptake, whereas delayed information provision may be associated with missed opportunities for cascade testing and subsequent risk stratification, enabling potentially life-saving preventive interventions [5, 6]. Repeated provision of information throughout the care pathway may help mitigate emotional burden as a barrier to genetic counseling and testing [5, 21].

The observed increase in uptake of genetic counseling over the study period, together with a trend toward shorter waiting times in later years, may reflect gradual improvements in awareness, service availability, and coordination between gynecologic oncology and genetics services [4, 11]. In addition, the small sample size in 2021 (*n* = 16) may have contributed to variability in uptake rates. However, the early phase of the study period coincided with the COVID-19 pandemic, which may have affected referral patterns, healthcare access, and waiting times for specialized consultations [32, 33]. Furthermore, recall bias may have influenced patient-reported perceptions regarding waiting times, particularly among participants treated during earlier study years and at different stages of the treatment trajectory [9].

Socioeconomic factors including family history of breast and/or ovarian cancer, higher educational background, parity, employment status and non-native speakers has been shown as indicators for genetic counseling and testing [34–38].

In line with previous findings, our data demonstrated that patients with a history of breast and/or ovarian cancer among relatives was a strong predictor of referral and utilization of genetic counseling among patients with ovarian cancer. This could reflect a higher motivation to pursue risk clarification for relatives, but it may also be influenced by a higher clinician driven information provision to this group [6, 34].

Educational background has been shown to be significantly associated with the uptake of genetic services, with higher educational attainment linked to more awareness of genetic testing and higher referral rates. This pattern is consistent with broader evidence indicating that health literacy and educational factors influence engagement with specialized preventive services [25, 35].

The association between parity and uptake of genetic counseling has been discussed controversially in the literature. Some studies showed that having children increases motivation to pursue genetic testing due to concerns regarding familial risk [4], whereas other cohort studies such as the GRACE Study by White et al. have not demonstrated a significant association [36]. Our findings support the relevance of parental status in decision-making regarding the utilization of genetic services.

Contrary to previous findings, where employment status and non-native speakers seem to negatively influence the proportion undergoing genetic testing, our data did not see this association [37, 38]. The absence of such association in our study may reflect uniform access to specialized oncologic and genetic services within the German healthcare system.

Strengths of our study include the integration of patient-reported outcomes with clinical data and the application of a structured problem frequency approach to summarize barriers across different domains of care. However, several limitations should be considered. The single-center design may limit generalizability of the findings to other healthcare systems and populations. The use of a non-validated study-specific questionnaire may limit comparability of results with other cohorts. In addition, as genetic counseling uptake was assessed only among questionnaire respondents, response bias cannot be excluded, and the observed uptake rates may not fully reflect those of the entire eligible study population. Although responders and non-responders showed comparable clinical characteristics, differential participation related to interest in genetic counseling, previous counseling experience, or attitudes toward genetic testing cannot be excluded. Furthermore, self-reported data are susceptible to recall bias, particularly regarding earlier phases of the study. Finally, findings from a university-based tertiary referral center may not fully reflect access patterns in non-specialized healthcare settings.

From a clinical perspective, our findings underscore the need for standardized, proactive referral approaches for genetic counseling in ovarian cancer care. Reliance on individual provider practices or patient self-initiation may be insufficient to ensure consistent access to genetic services. Standardized referral structures and proactive integration of genetic counseling into oncologic care may represent potential strategies to address organizational barriers and facilitate more consistent access to genetic services [39, 40].

Our findings further highlight the broader challenge of translating recommendations for universal genetic assessment into routine clinical practice. Variability in genetic counseling and testing uptake has been reported across different healthcare systems, indicating that implementation remains incomplete despite broad recommendations for genetic assessment [26, 28]. Structural and organizational factors, including provider referral, information provision, and access to genetic services, may contribute to these implementation gaps [4, 28].

In addition, the observation that a subset of patients who had not previously attended genetic counseling reconsidered genetic services after completing the questionnaire suggests that repeated patient engagement may prompt renewed consideration of genetic counseling and highlights the potential value of structured information tools [41]. However, no longitudinal follow-up was predefined within the study protocol to assess whether this reconsideration translated into subsequent uptake of genetic counseling. This warrants further evaluation in prospective studies.

In line with quality improvement approaches used in in oncology care services, the problem frequency framework may represent a useful tool for quality improvement by enabling healthcare providers and institutions to identify and prioritize problem domains along the care pathway [42].

Future studies should assess structured referral pathways, integrated care models, and repeated patient engagement approaches to improve access to genetic counseling in ovarian cancer care.

## Conclusion

Despite recommendations for universal genetic counseling in ovarian cancer care, gaps in implementation remain evident in routine clinical practice. Organizational and informational barriers represent prominent obstacles to uptake. Our findings highlight the potential value of standardized referral pathways, proactive information provision, and repeated patient engagement to facilitate access to genetic services. Further multicenter studies are warranted to evaluate these strategies across different healthcare settings.

## Supplementary Information

Supplementary Material 1.

## Data Availability

All data generated or analyzed during this study are contained within the article. Further enquiries can be directed to the corresponding author.
