# Supplementary material for: Bridging the gap between guidelines and practice: genetic counseling uptake and barriers in patients with ovarian malignancies
Source: BMC Womens Health. 2026 Sep 29;26:482. doi: 10.1186/s12905-026-04940-y (PMC13625251; doi:10.1186/s12905-026-04940-y)
Supplement: Supplementary file 1 [file 12905_2026_4940_MOESM1_ESM.zip › translated Questionnaire.pdf]

UNIVERSITÄTS KLINIKUM HEIDELBERG

English Translation of the Original German Questionnaire

Study Title

Uptake of Genetic Counseling Among Patients with Ovarian Cancer at Heidelberg University Hospital: A Follow-up Survey

Please answer the following questions as accurately as possible. All responses are anonymous and will be used exclusively for scientific research.

**1. Information about Genetic Counseling and Genetic Testing**	
**1.1 Were you informed about the recommendation for genetic counseling during your treatment at Heidelberg University Hospital or the National Center for Tumor Disease (NCT)?**	O Yes<br>O No<br>O I do not remember
**1.2. If yes, when were you first informed?**<br>[Please select one answer]	O Before surgery<br>O After surgery<br>O During chemotherapy<br>O After completion of chemotherapy<br>O At recurrence or disease progression<br>O At another time (Please specify): ________________<br>O I do not remember
**1.3. If no, did someone else inform you about the recommendation for genetic counseling?**	O No<br>O Yes: My community gynecologist<br>O Yes: My general practitioner<br>O Yes: Another healthcare professional<br>O I do not remember
**2. Uptake of Genetic Counseling**	
**2.1. Did you attend genetic counseling?**	O Yes<br>O No<br>O I do not remember
**2.2. If yes, what was the main reason for attending genetic counseling?**<br>[Please select one answer]	O Recommendation by a physician at Heidelberg University Hospital<br>O Recommendation by the NCT or my oncologist<br>O Recommendation by my community gynecologist<br>O Recommendation by my general practitioner<br>O Recommendation by relatives or friends<br>O Other (Please specify): ________________________<br>O I do not remember
**2.3. If yes, who arranged your appointment?**<br>[Please select one answer]	O Hospital physician<br>O National Center for Tumor Disease (NCT)<br>O My community gynecologist<br>O My general practitioner<br>O I arranged it myself<br>O A family member or a friend<br>O Other (Please specify): ________________________<br>O I do not remember
**2.4. If yes, where did you receive genetic counseling?**	O Institute of Human Genetics, Heidelberg University Hospital<br>O Another genetic counseling Centre
**2.5. If yes, have you proceeded with germline genetic testing?**	O Yes<br>O No<br>O I do not remember
**2.6. If yes, how did you get to your first appointment?**<br>[Please select one answer]	O Public transport<br>O Taxi<br>O Private car<br>O Other (Please specify): ________________________<br>O I do not remember

**2.7. If no, why you did not attend genetic counseling?**	O I was concerned about the emotional impact of the test results<br>O I was concerned about the impact of the test results on my family<br>O I was not interested/ I decided against it<br>O I was not informed about genetic counseling or not offered to me<br>O I was unable to obtain an appointment<br>O Waiting time for an appointment was too long<br>O Organizational difficulties (e.g., travel distance)<br>O Other (Please specify): ________________________
**2.8. If no, would you consider attending genetic counseling after receiving this questionnaire?**	O Yes<br>O No<br>O I am not sure
**3. Improving Genetic Counseling**	
**3.1. Please rank the following factors according to their importance in your decision to undergo genetic counseling**<br><br>[Assign each factor a number from 1 to 7]<br>1= “Most important “<br>7= “Least important “	[ ] Recommendation by physicians<br>[ ] Recommendation by relatives or friends<br>[ ] Previous personal experience<br>[ ] Information from the social media or internet<br>[ ] Short waiting time for an appointment<br>[ ] Other (Please specify): ________________
**3.2. How would you rate the information you received about genetic counseling?**<br>[Please select one number]	**Please rate your experience on a scale from 1 to 10, where:**<br>**1= Very poor**<br>**10= Excellent**<br>**Scale 1= “very poor “to 10= “Excellent “)**<br>**1** O **2** O **3** O **4** O **5** O **6** O **7** O **8** O **9** O **10** O
**3.3. How would you rate the waiting time for your appointment?**<br>[Please select one number]	**Please rate your experience on a scale from 1 to 10, where:**<br>**1= Very long**<br>**10= Very short**<br>**Scale 1= “very long “to 10= “very short “)**<br>**1** O **2** O **3** O **4** O **5** O **6** O **7** O **8** O **9** O **10** O
**3.4. What additional information would you have liked to received?**	(Please specify): _______________________
**4. Demographic Information**	
**4.1. Employment status at the time of diagnosis**	O Full-time<br>O Part-time<br>O Unemployed<br>O Retired<br>O Student/In education<br>O Other (Please specify): ________________
**4.2. Highest educational qualification**	O Primary or Intermediate school<br>O Secondary school<br>O University entrance qualification (Abitur or equivalent)<br>O University degree<br>O Doctoral degree (PhD/MD)<br>O Other (Please specify): ________________
**4.3. Do you have children?**<br>**Geschlecht der Kinder**<br>**Wie alt?**	O Yes (O Female O Male) O No<br>O Number of children: _______<br>O Age(s): _______

**4.4. Native language**	O German<br>O Other (Please specify): _________________
**4.5. Do you have a family history of breast and/or ovarian cancer?**	O Yes O No O I do not know<br>O If yes, please specify the affected relative(s):<br>_________________
**Additional Comments**	
Please use the space below for any additional comments, suggestions or criticism regarding genetic counseling or this survey.	

Thank you for participating!

Your responses will help improve access to genetic counseling and optimize patient care for women with ovarian cancer at Heidelberg University Hospital.

As the next step, please place the completed questionnaire and the signed informed consent in the enclosed pre-addressed return envelope and mail it back to us. Postage is prepaid.
