# Supplementary material for: Bridging the gap between guidelines and practice: genetic counseling uptake and barriers in patients with ovarian malignancies
Source: BMC Womens Health. 2026 Sep 29;26:482. doi: 10.1186/s12905-026-04940-y (PMC13625251; doi:10.1186/s12905-026-04940-y)
Supplement: Supplementary file 1 [file 12905_2026_4940_MOESM1_ESM.zip › Revised_Questionnaire.docx]

**English Translation of the Original German Questionnaire**

**Study Title**

**Uptake of Genetic Counseling Among Patients with Ovarian Cancer at Heidelberg University Hospital: A Follow-up Survey**

Please answer the following questions as accurately as possible. All responses are anonymous and will be used exclusively for scientific research.

| **1. Information about Genetic Counseling and Genetic Testing** | |
| --- | --- |
| **1.1 Were you informed about the recommendation for genetic counseling during your treatment at Heidelberg University Hospital or the National Center for Tumor Disease (NCT)?** | O Yes  O No  O I do not remember |
| **1.2. If yes, when were you first informed?**  [Please select one answer] | O Before surgery  O After surgery  O During chemotherapy  O After completion of chemotherapy  O At recurrence or disease progression  O At another time (Please specify): ________________  O I do not remember |
| **1.3. If no, did someone else inform you about the recommendation for genetic counseling?** | O No  O Yes: My community gynecologist  O Yes: My general practitioner  O Yes: Another healthcare professional  O I do not remember |
| **2. Uptake of Genetic Counseling** | |
| **2.1. Did you attend genetic counseling?** | O Yes  O No  O I do not remember |
| **2.2. If yes, what was the main reason for attending genetic counseling?**  [Please select one answer] | O Recommendation by a physician at Heidelberg University Hospital  O Recommendation by the NCT or my oncologist  O Recommendation by my community gynecologist  O Recommendation by my general practitioner  O Recommendation by relatives or friends  O Other (Please specify): ________________________  O I do not remember |
| **2.3. If yes, who arranged your appointment?**  [Please select one answer] | O Hospital physician  O National Center for Tumor Disease (NCT)  O My community gynecologist  O My general practitioner  O I arranged it myself  O A family member or a friend  O Other (Please specify): ________________________  O I do not remember |
| **2.4. If yes, where did you receive genetic counseling?** | O Institute of Human Genetics, Heidelberg University Hospital  O Another genetic counseling Centre |
| **2.5. If yes, have you proceeded with germline genetic testing?** | O Yes  O No  O I do not remember |
| **2.6. If yes, how did you get to your first appointment?**  [Please select one answer] | O Public transport  O Taxi  O Private car  O Other (Please specify): ________________________  O I do not remember |
| **2.7. If no, why you did not attend genetic counseling?** | O I was concerned about the emotional impact of the test results  O I was concerned about the impact of the test results on my family  O I was not interested/ I decided against it  O I was not informed about genetic counseling or not offered to me  O I was unable to obtain an appointment  O Waiting time for an appointment was too long  O Organizational difficulties (e.g., travel distance)  O Other (Please specify): ________________________ |
| **2.8. If no, would you consider attending genetic counseling after receiving this questionnaire?** | O Yes  O No  O I am not sure |
| **3. Improving Genetic Counseling** | |
| **3.1. Please rank the following factors according to their importance in your decision to undergo genetic counseling**  [Assign each factor a number from 1 to 6]  1= “Most important “  6= “Least important “ | \| Recommendation by physicians; \| Recommendation by relatives or friends; \| Previous personal experience; \| Information from the social media or internet; \| Short waiting time for an appointment; \| Other (Please specify): ________________ |
| **3.2. How would you rate the information you received about genetic counseling?**  [Please select one number] | **Please rate your experience on a scale from 1 to 10, where:** **1= Very poor** **10= Excellent** **Scale 1= “very poor “to 10= “Excellent “)** **1** \| **2** \| **3** \| **4** \| **5** \| **6** \| **7** \| **8** \| **9** \| **10**; O \| O \| O \| O \| O \| O \| O \| O \| O \| O |
| **3.3. How would you rate the waiting time for your appointment?**  [Please select one number] | **Please rate your experience on a scale from 1 to 10, where:** **1= Very long** **10= Very short** **Scale 1= “very long “to 10= “very short “)** **1** \| **2** \| **3** \| **4** \| **5** \| **6** \| **7** \| **8** \| **9** \| **10**; O \| O \| O \| O \| O \| O \| O \| O \| O \| O |
| **3.4. What additional information would you have liked to received?** | (Please specify): ______________________ |
| **4. Demographic Information** |  |
| **4.1. Employment status at the time of diagnosis** | O Full-time  O Part-time  O Unemployed  O Retired  O Student/In education  O Other (Please specify): ________________ |
| **4.2. Highest educational qualification** | O Primary or Intermediate school  O Secondary school  O University entrance qualification (Abitur or equivalent)  O University degree  O Doctoral degree (PhD/MD)  O Other (Please specify): ________________ |
| **4.3. Do you have children?** | O Yes (O Female O Male) O No  O Number of children: _______  O Age(s): _______ |
| **4.4. Native language** | O German  O Other (Please specify): ________________ |
| **4.5. Do you have a family history of breast and/or ovarian cancer?** | O Yes O No O I do not know  O If yes, please specify the affected relative(s):  ________________ |
| **Additional Comments** | |
| Please use the space below for any additional comments, suggestions or criticism regarding genetic counseling or this survey. | |

**Thank you for participating!**

Your responses will help improve access to genetic counseling and optimize patient care for women with ovarian cancer at Heidelberg University Hospital.

**As the next step, please place the completed questionnaire and the signed informed consent in the enclosed pre-addressed return envelope and mail it back to us. Postage is prepaid.**
